# Effect of the Characteristic Size and Content of Graphene on the Crack Propagation Path of Alumina/Graphene Composite Ceramics

**DOI:** 10.3390/ma14030611

**Published:** 2021-01-28

**Authors:** Benshuai Chen, Guangchun Xiao, Mingdong Yi, Jingjie Zhang, Tingting Zhou, Zhaoqiang Chen, Yongpeng Zhang, Chonghai Xu

**Affiliations:** 1 School of Mechanical and Automotive Engineering, Qilu University of Technology (Shandong Academyof Sciences), Jinan 250353, China; benshuaichen@163.com (B.C.); new-raul@163.com (M.Y.); zjj@qlu.edu.cn (J.Z.); zhoutingting506@163.com (T.Z.); czq@qlu.edu.cn (Z.C.); researcher_cbs@163.com (Y.Z.); xch@qlu.edu.cn (C.X.); 2 Key Laboratory of Advanced Manufacturing and Measurement and Control Technology for Light Industry in Universities of Shandong, Qilu University of Technology (Shandong Academy of Sciences), Jinan 250353, China

**Keywords:** graphene, composite ceramic tool material, crack propagation, toughening mechanism

## Abstract

In this paper, the Voronoimosaic model and the cohesive element method were used to simulate crack propagation in the microstructure of alumina/graphene composite ceramic tool materials. The effects of graphene characteristic size and volume content on the crack propagation behavior of microstructure model of alumina/graphene composite ceramics under different interfacial bonding strength were studied. When the phase interface is weak, the average energy release rate is the highest as the short diameter of graphene is 10–50 nm and the long diameter is 1600–2000 nm. When the phase interface is strong, the average energy release rate is the highest as the short diameter of graphene is 50–100 nm and the long diameter is 800–1200 nm. When the volume content of graphene is 0.50 vol.%, the average energy release rate reaches the maximum. When the velocity load is 0.005 m s^−1^, the simulation result is convergent. It is proven that the simulation results are in good agreement with the experimental phenomena.

## 1. Introduction

Ceramic materials have attracted attention because of their high hardness and wear resistance [1,2,3], and alumina ceramics are the most widely used thanks to their better oxidation resistance and low price [4]. However, pure alumina is very brittle, and it is prone to fracture [5,6]. In order to improve the toughness of pure alumina ceramic materials, reinforcing phases are usually added into ceramic materials. The reinforcing phases are usually boride [7], oxide [8], carbide [9], and so on.

Graphene has a high specific surface area and can be closely combined with ceramic matrix, which can improve the fracture toughness of ceramic materials [10,11,12]. In recent years, graphene toughened ceramic composite materials have been widely studied [13,14]. For example, Wang et al. [15] added WC, TiC, and graphene into Al_2_O_3_ matrix to obtain Al_2_O_3_/WC/TiC/graphene composite ceramic tool materials, whose optimal indentation fracture toughness, Vickers hardness, and flexural strength reached 9.42 MPa.m^0.5^, 24.64 GPa, and 646.31 MPa, respectively. Ahmad et al. [16] used nano zirconia and graphene nano-sheets to toughen alumina ceramic composite, and it was reported that the fracture toughness increased by 155% and the microhardness increased by 17% compared with the monolithic alumina ceramic.

Nagaraj et al. [17] found that fracture and pull-out of graphene would lead to more crack deflection and crack branching. The study by Yin et al. [18] showed that the network structure formed by graphene in the ceramic matrix can enhance the interface bonding, leading to more crack bridging and deflection. At present, the macro toughening mechanisms of graphene have been mostly studied, such as crack deflection, graphene pull-out, and graphene fracture [19,20,21], while the micro and nano interface toughening mechanism of graphene is seldom studied. The graphene characteristic size [22], interface structure [23], and interface bonding strength [24] play an important role in improving the mechanical properties of graphene toughened ceramic materials. Therefore, it is of great significance to study the toughening mechanism of micro-interface and nano-interface by establishing the mechanical model of the interface between graphene and ceramic matrix.

The finite element method (FEM) is a numerical analysis method based on variational and interpolation principles [25,26,27]. The Voronoi tessellation is composed of some polygons similar to the material grain geometry, which can be used to characterize the microstructure of ceramic materials [28,29]. Zhou et al. [30] characterized the microstructure of single-phase alumina ceramic tool materials by Voronoi tessellation, and made a series of simulations related to crack propagation. Cohesion refers to the interaction between atoms or molecules in matter. The cohesion element method can be used to simulate the fracture behavior of materials [31].

In previous studies, the microstructure model of alumina/graphene composite ceramics was established by the inserting cohesion units, and the influence of interfacial bonding strength between alumina and graphene on the crack propagation behavior was studied. Based on previous studies, this paper studies the effects of graphene particle size, graphene content, and velocity load on the crack propagation path, and compares them with the experimental results.

## 2. Materials and Methods

**Figure 1:**
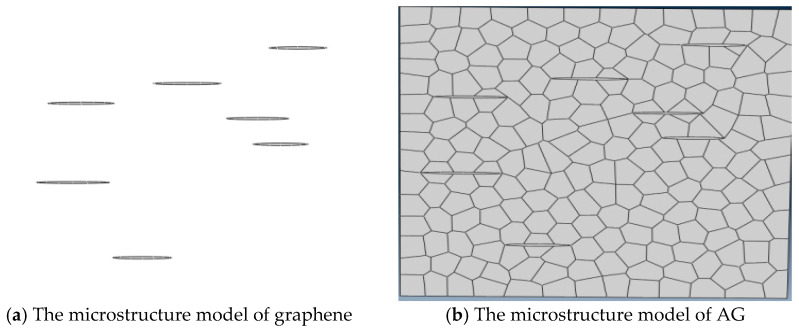
Microstructure model of composite ceramic tool material [32]. AG, alumina/graphene composite ceramics.

The overall size of the Voronoi mesh model established in this paper is 10 μm × 10 μm, the number of grains is about 160, and the average grain diameter is about 0.8 μm. On the basis of obtaining the Voronoi mesh to characterize the microstructure of single-phase Al_2_O_3_ ceramic tool material, the microstructure of graphene was characterized by ellipse. It is necessary to design the size of the long diameter and short diameter of the ellipse, and then characterize the graphene microstructure by randomly generating the corresponding size of the ellipse in the same 10 μm × 10 μm square using Python scripting language. The microstructure model of graphene is shown in Figure 1a. The microstructure model of alumina/graphene (AG) composite ceramic tool material is shown in Figure 1b. Apply symmetrical velocity load on both sides of the model. In order to form a stress concentration, the initial crack is usually placed in the middle position of the left side of the model. The microtissue model of AG composite ceramic tool material is divided into triangle grid, and the inner cohesion unit is inserted in the adjacent triangle unit [32].

In our previous work, the influence of interfacial bonding strength on the crack propagation path of AG composite ceramics was studied. The results show that the toughening effect is best when the cohesion parameters of alumina grain boundary and intragranular are taken as the cohesion parameters of alumina/graphene phase interface [32]. Therefore, the cohesion parameters of alumina/graphene phase interface are set as the cohesion parameters of the alumina grain boundary (weak interface) and alumina grain interior (strong interface), respectively. The cohesion parameters of the microstructure model of AG materials are shown in Table 1.

The average energy release rate is used to characterize the fracture toughness of composite ceramics [33]. In this study, the explicit integration algorithm is adopted, which needs to apply the velocity load, but the velocity load will have a certain impact on the simulation results of the crack growth. In order to reduce this effect, the relationship between the model and the velocity load can be expressed by the strain rate [34]:

(1) $$\varepsilon \;=\;\frac{V}{H}$$

where *ε* represents the strain rate. *V* represents the velocity load, which is applied to the upper and lower sides of the model. *H* represents the half of the total height in the model. Relevant research [35] shows that the load strain rate applied by the fracture behavior analysis model of ceramic tool materials is generally kept between 1 × 10^3^ S^−1^ and 1 × 10^5^ S^−1^. For this model, the speed range of the strain rate is between 0.005 m s^−^^1^ and 0.05 m s^−^^1^. However, the smaller the value of velocity load, the longer the time needed for crack propagation simulation calculation [33]. Therefore, the value of velocity load should be selected reasonably on the premise of ensuring the calculation accuracy.

## 3. Results and Discussion

### 3.1. Effect of the Characteristic Size of Graphene on the Crack Propagation Path of AG Composite Ceramics

The effects of the short diameter and long diameter of graphene on the crack propagation path were studied, respectively. The specific data are shown in Table 2. The volume content of graphene is 0.75 vol.%, and the initial crack length is about 0.8 μm, preset on the left side of the model.

#### 3.1.1. Effect of the Short Diameter of Graphene

**Figure 2:**
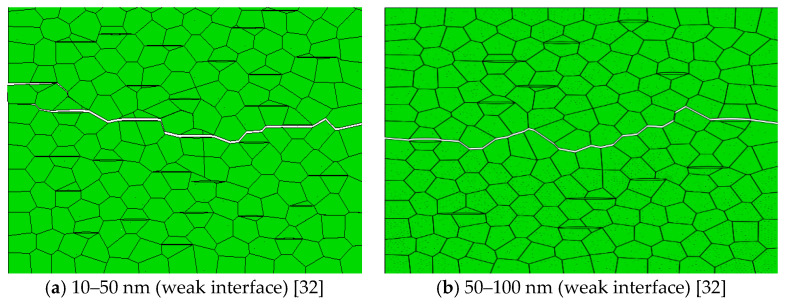

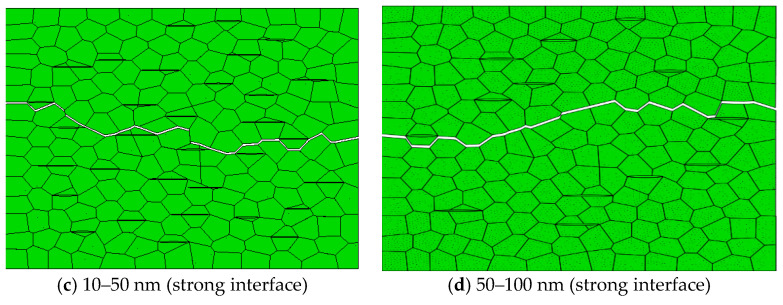
The simulation results of crack propagation paths with different short diameters when the long diameter of graphene is 800–1200 nm.

The long diameter of graphene is set as 800–1200 nm, and the short diameters are set as 10–50 nm, 50–100 nm, 100–150 nm, and 150–200 nm, respectively. The simulation results of the crack propagation are shown in Figure 2a,b when the phase interface is weak. Cracks all propagate along graphene and, compared with Figure 2b, the crack propagation path in Figure 2a is more tortuous. The reason is that, under the same volume content, the number of graphene grains with a short diameter of 10–50 nm is more, and it is easier to produce crack branches. It can be seen from Table 3 that the energy dissipation and crack propagation length of graphene with short diameter of 10–50 nm are the largest. With the increase of the short diameter of graphene, the average energy release rate shows a downward trend, because microcracks can alleviate the stress concentration near the main crack tip [33,34].

When the phase interface is weak, the smaller the short diameter of graphene, the more obvious the toughening effect. When the short diameter of graphene is 10–50 nm, the resistance to crack is the strongest.

The simulation results of the crack propagation path are shown in Figure 2c,d when the phase interface is strong. Figure 2c shows the simulation results when the short diameter of graphene is 10–50 nm. Compared with Figure 2d, the crack propagation path is tortuous. The simulation results are shown in Figure 2d when the short diameter of graphene is 50–100 nm. The cracks deflect along some high-strength interfaces and consume some energy. It can be seen from Table 3 that, with the increase of the short diameter of graphene, the average energy release first increases and then decreases. When the short diameter of graphene is 50–100 nm, the average energy release rate is highest and the toughening effect is obvious.

When the phase interface is weak, the smaller the short diameter of graphene, the more obvious the toughening effect. When the phase interface is strong, the toughening effect is relatively good when the short diameter graphene is 50–100 nm. According to the above analysis, the short diameter sizes of graphene are determined to be 10–50 nm and 50–100 nm.

#### 3.1.2. Effect of the Long Diameter of Graphene

When the short diameter is 10–50 nm or 50–100 nm, and the long diameter is 800–1200 nm, 1200–1600 nm, 1600–2000 nm, and 2000–2400 nm, respectively, the crack propagation at different interface strengths is simulated.

**Figure 3:**
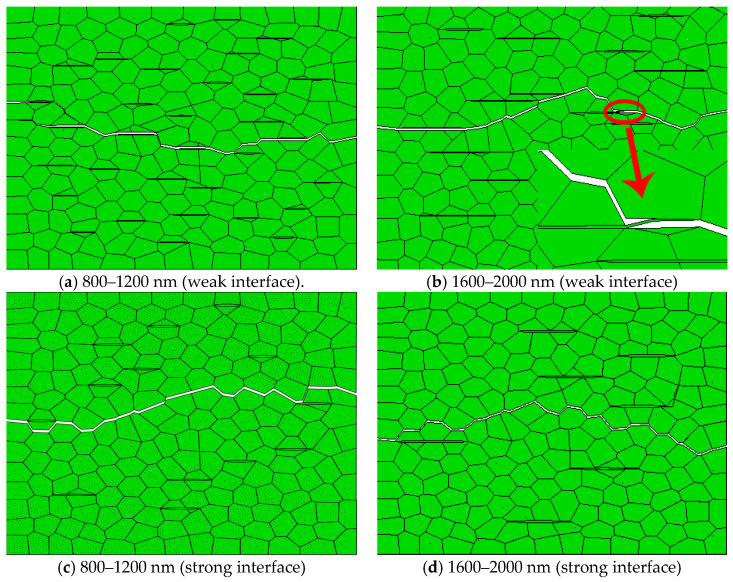
The simulation results of crack propagation paths with different long diameters when the short diameter of graphene is 10–50 nm.

The simulation results of crack propagation paths with different long diameters are shown in Figure 3a,b when the short diameter of graphene is 10–50 nm and the phase interface is weak. The simulation results of graphene with the long diameter of 800–1200 nm are shown in Figure 3a. The crack propagation path is tortuous and the crack propagation length is large. The simulation results are shown in Figure 3b when the long diameter of graphene is 1600–2000 nm. When cracks encounter the large graphene in the process of propagation, it is difficult to penetrate the high-strength graphene and form crack bridging, which is beneficial to improve the fracture toughness of materials. It can be seen from Table 4 that the crack propagation length of graphene decreases at first and then increases with the increase of its long diameter. An excessively long diameter of graphene will weaken the material. When the short diameter of graphene is 10–50 nm and the phase interface is weak, the average energy release rate is higher when the long diameter of graphene is 1600–2000 nm, and the toughening effect is better.

The simulation results of crack propagation paths with different long diameters are shown in Figure 3c,d when the short diameter of graphene is 10–50 nm and the phase interface is strong. The crack propagates first along the grain boundary of ceramic, which is a typical intergranular fracture mode [5,34]. When the initial crack meets graphene, it may deflect or staple at the grain boundary, and then re-crack at other weak areas owing to the strong interfacial bond between graphene and the ceramic matrix. A high strength interface usually consumes a lot of energy, but compared with weak interface bonding, the energy release rate of strong interface bonding is not high (Table 4). The possible reason is that excessive crack deflection reduces the energy release at the strong interface. It can be seen from Table 4 that, when the long diameter of graphene is 800–1200 nm, the average energy release rate is higher. From the above analysis, it can be seen that the toughening methods of graphene are mainly crack deflection, crack bridging, and crack branching. When the phase interface is weak, the average energy release rate is the highest under the conditions that the short diameter of graphene is 10–50 nm and the long diameter is 1600–2000 nm. When the phase interface is strong, the average energy release rate is the highest under the conditions that the short diameter of graphene is 50–100 nm and the long diameter is 800–1200 nm.

### 3.2. Effect of Graphene Volume Content on the Crack Propagation Path

The effect of graphene volume content on the crack propagation path of AG composites was simulated under different microstructures (Table 5).

**Figure 4:**
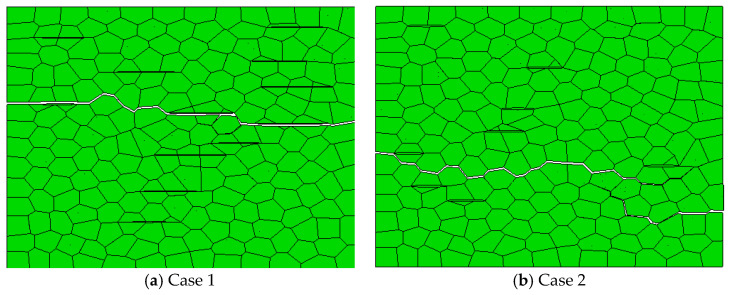
The simulation results of crack propagation when the volume content of graphene is 0.50 vol.%.

**Figure 5:**
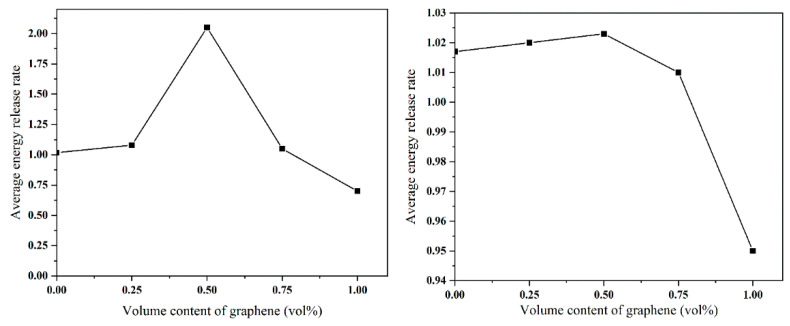
The average energy release rate varies with the volume content of graphene.

The simulation results of crack propagation at weak interface are shown in Figure 4a. Cracks mainly propagate along graphene, but with the increase of graphene volume content, crack bridging appears. It is difficult to penetrate the high strength graphene when the crack encounters the longer graphene in the process of growth. Thus, new cracks are generated on the other side of the graphene to form crack bridging. It can be seen from Table 6 and Figure 5a that the average energy release rate increases first and then decreases with the increase of graphene volume content. This is probably because too much graphene weakens the material. When the volume content of graphene is 0.50 vol.%, the average energy release rate reaches the maximum.

The simulation results of crack propagation of each microscopic model under the strong interface are shown in Figure 4b. The crack mainly deflects around graphene, and with the increase of graphene volume content, the crack propagation path becomes more and more tortuous. As shown in Figure 4b, the crack does not completely penetrate through the material, but forms secondary cracks at other weak positions (because of the strong interfacial bonding force, the crack propagation is hindered). It can be seen from Table 6 and Figure 5 that, with the increase of graphene volume content, the average energy release rate shows a trend of first rising and then falling. The optimum volume content of graphene is 0.5 vol.%.

### 3.3. Effect of the Velocity Load

**Figure 6:**
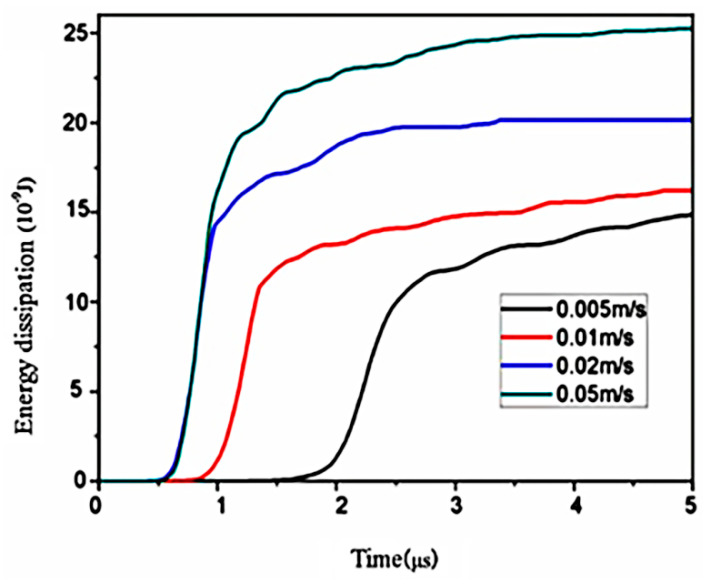
Energy dissipation under different velocity loads.

The law of the energy dissipation under different velocity loads is shown in Figure 6. With the increase of the velocity load, the energy dissipation also increases, because more secondary cracks are formed. The greater the velocity load, the more secondary cracks. When the velocity load is 0.005 m s^−1^, the crack begins to form at about 2 μs. With the increase of velocity load, the time of crack generation is gradually advanced. When the velocity load is 0.005 m s^−1^ and 0.01 m s^−1^, the crack propagation path is a single crack propagation and a few secondary cracks, and the curve at this stage is relatively stable. In general, the velocity load of 0.005 m s^−1^ in this study can ensure the convergence of simulation calculation.

According to the above research results, with the increase of graphene volume content, the average energy release rate of materials showed a trend of increasing at first and then decreasing. When the volume content of graphene is 0.50 vol.%, the average energy release rate reaches the maximum. This is different from that reported by Meng et al. [36] that the fracture toughness is best when the volume content of graphene is 0.75 vol.%. The reason is that graphene is a multi-layer material, and there are large gaps between the layers. In the simulation process, the multi-layer graphene is regarded as a whole and the influence of porosity and other factors is ignored, which has a certain impact on the simulation results.

**Figure 7:**
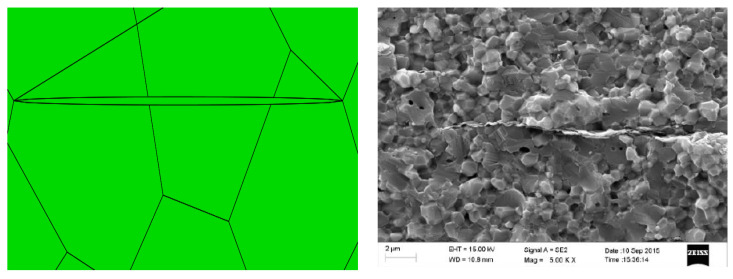
The microstructure of AG composite ceramic tool material [36].

The graphene sheets in the alumina/graphene composite ceramic cutting tool materials reported by Meng et al. [36] of our research group show an obvious parallel relationship (as shown in Figure 7), and the grain size of Al_2_O_3_ near graphene is smaller than that far away from graphene. The grain size of Al_2_O_3_ is about 0.8 μm, which is consistent with the model established in this paper.

**Figure 8:**
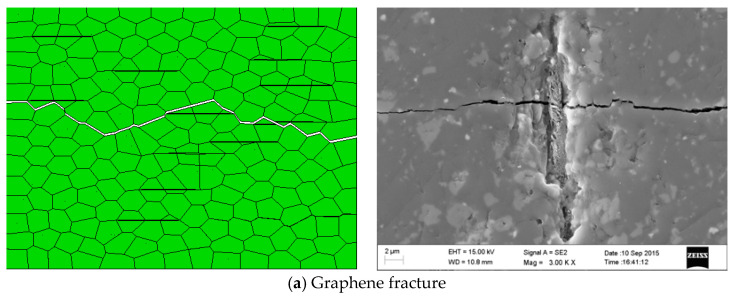

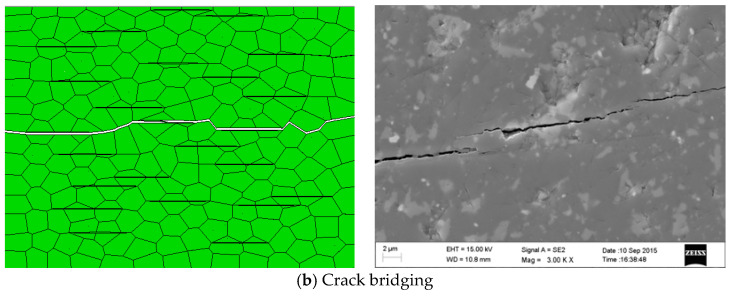
Common toughening mechanism of graphene in Al_2_O_3_ matrix [36].

Through the simulation and analysis, the toughening modes such as crack deflection, graphene fracture, and crack bridging are observed in the composite ceramic tool material. As shown in Figure 8a, the graphene sheet is tightly bonded with the matrix, and graphene is broken by cracks, which consumes a lot of fracture energy. In Figure 8b, because of the uneven dispersion of graphene, the bonding between the graphene sheet and matrix is not particularly tight, thus forming crack bridging. The simulation results are consistent with the previous experimental results of the research group, which shows that the model established in this paper is feasible.

## 4. Conclusions

In this paper, the Voronoimosaic model and the cohesive element method were used to simulate crack propagation in the microstructure of alumina/graphene composite ceramic tool materials. The effects of graphene characteristic size, graphene content, and loading speed were studied. The conclusions are as follows:

(1) When the phase interface is weak, the average energy release rate is the highest when the short diameter of graphene is 10–50 nm and the long diameter is 1600–2000 nm. When the phase interface is strong, the average energy release rate is the highest when the short diameter of graphene is 50–100 nm and the long diameter is 800–1200 nm.

(2) With the increase of graphene volume content, the average energy release rate increased first and then decreased. The average energy release is the largest when the content of graphene is 0.50 vol.%.

(3) The results show that the convergence of the simulation calculation can be ensured when the velocity load is 0.005 m s^−1^. It is proven that the simulation results are in good agreement with the experimental results.

## Figures and Tables

**Table 1:** The cohesive parameters of the microstructure model of alumina/graphene composite ceramics (AG) [32].

Interfacial Bonding Strength	Tmax (MPa)	Γ (J m^−2^)	K (Mpa mm^−1^)
weak interface	186	1	1.73 × 10^9^
strong interface	644	2.3	9 × 10^9^

**Table 2:** The characteristic size of graphene.

The Long Diameter of Graphene (nm)	The Short Diameter of Graphene (nm)
800–1200	10–50
1200–1600	50–100
1600–2000	100–150
2000–2500	150–200

**Table 3:** The calculation results of microscopic models with different short diameters when the long diameter of graphene is 800–1200 nm.

The Short Diameter of Graphene (nm)	Energy Dissipation (10^−9^ J)		Crack Propagation Length (μm)		Average Energy Release Rate	
	Weak Interface	Strong Interface	Weak Interface	strong interface	Weak Interface	Strong Interface
10–50	16.5	17.2	15.2	16.7	1.07	1.02
50–100	14.7	16.9	14.09	16.27	1.042	1.039
100–150	15	18	14.46	17.625	1.037	1.021
150–200	14.7	22.7	14.8642	22.185	0.989	1.02

**Table 4:** The calculation results of microscopic models with different long diameters when the short diameter of graphene is 10–50 nm.

The Short Diameter of Graphene (nm)	Energy Dissipation (10^−9^ J)		Crack Propagation Length (μm)		Average Energy Release Rate	
	Weak Interface	Strong Interface	Weak Interface	Strong Interface	Weak Interface	Strong Interface
800–1200	16.5	16.5	15.39	16.27	1.07	1.039
1200–1600	15	15	14.545	14.871	1.0312	1.015
1600–2000	14.2	18.4	13.446	17.747	1.05	1.036
2000–2400	15.3	19.8	16.38	18.7	0.934	1.05

**Table 5:** Microstructure types of AG composites.

Microstructure	Interfacial Bonding Strength	The Short Diameter of Graphene (nm)	The Long Diameter of Graphene (nm)
Case 1	weak interface	10–50	1600–2000
Case 2	strong interface	50–100	800–1200

**Table 6:** The calculation results of crack propagation under different graphene volume contents.

The Volume Content of Graphene (vol.%)	Energy Dissipation (10^−9^ J)		Crack Propagation Length (μm)		Average Energy Release Rate	
	Case 1	Case 2	Case 1	Case 2	Case 1	Case 2
0.25	15.3	15.6	14.1	15.29	1.08	1.02
0.5	31.2	18.9	15.2	18.47	2.05	1.023
0.75	14.2	16.5	13.4	16.27	1.05	1.01
1	13.9	18.7	19.8	19.53	0.70	0.95

## Data Availability

Data sharing is not applicable to this article.
